# A 3D Bioprinted Material That Recapitulates the Perivascular Bone Marrow Structure for Sustained Hematopoietic and Cancer Models

**DOI:** 10.3390/polym13040480

**Published:** 2021-02-03

**Authors:** Caitlyn A. Moore, Zain Siddiqui, Griffin J. Carney, Yahaira Naaldijk, Khadidiatou Guiro, Alejandra I. Ferrer, Lauren S. Sherman, Murat Guvendiren, Vivek A. Kumar, Pranela Rameshwar

**Affiliations:** 1 Department of Medicine, Rutgers New Jersey Medical School, 185 South Orange Avenue, Newark, NJ 07103, USA; cam618@gsbs.rutgers.edu (C.A.M.); gcarney@bu.edu (G.J.C.); yn135@njms.rutgers.edu (Y.N.); guirokh@njms.rutgers.edu (K.G.); aif25@gsbs.rutgers.edu (A.I.F.); shermala@njms.rutgers.edu (L.S.S.); 2 Department of Medicine, Rutgers School of Graduate Studies, New Jersey Medical School, 185 South Orange Avenue, Newark, NJ 07103, USA; 3 Department of Biomedical Engineering, New Jersey Institute of Technology, 323 Martin Luther King Jr. Blvd, Newark, NJ 07102, USA; zs67@njit.edu (Z.S.); muratg@njit.edu (M.G.); vak@njit.edu (V.A.K.); 4 Department of Chemical, Biological and Pharmaceutical Engineering, New Jersey Institute of Technology, 323 Martin Luther King Jr. Blvd, Newark, NJ 07102, USA; 5 Department of Restorative Dentistry, Rutgers School of Dental Medicine, 110 Bergen St, Newark, NJ 07103, USA

**Keywords:** alginate, methylcellulose, hydrogel, bone marrow, bioprinting, breast cancer, stem cells

## Abstract

Translational medicine requires facile experimental systems to replicate the dynamic biological systems of diseases. Drug approval continues to lag, partly due to incongruencies in the research pipeline that traditionally involve 2D models, which could be improved with 3D models. The bone marrow (BM) poses challenges to harvest as an intact organ, making it difficult to study disease processes such as breast cancer (BC) survival in BM, and to effective evaluation of drug response in BM. Furthermore, it is a challenge to develop 3D BM structures due to its weak physical properties, and complex hierarchical structure and cellular landscape. To address this, we leveraged 3D bioprinting to create a BM structure with varied methylcellulose (M): alginate (A) ratios. We selected hydrogels containing 4% (*w*/*v*) M and 2% (*w*/*v*) A, which recapitulates rheological and ultrastructural features of the BM while maintaining stability in culture. This hydrogel sustained the culture of two key primary BM microenvironmental cells found at the perivascular region, mesenchymal stem cells and endothelial cells. More importantly, the scaffold showed evidence of cell autonomous dedifferentiation of BC cells to cancer stem cell properties. This scaffold could be the platform to create BM models for various diseases and also for drug screening.

## 1. Introduction

Translational medicine is propelled by a cyclical bench-to-bedside-to-bench approach in which laboratory data drives clinical strategies, generating questions for further investigation in the lab. However, this iterative process is largely driven by two-dimensional (2D) in vitro and/or preclinical animal models, which alone may not be sufficient for effective translation to the clinical arena. Drug approval rate by the US Food and Drug Administration (FDA) hovers around 10%, with ~54% failure during late-stage clinical development [1,2,3]. In order to improve scientific translational efficiency, current research and development approaches must evolve to mimic the three-dimensional (3D) organ structures. Indeed, in recent years, 3D bioprinting has emerged as a particularly powerful technique to create highly specialized and customizable 3D in vitro systems for the purposes of tissue modeling and regeneration [4].

The incorporation of 3D in vitro platform has improved research to evaluate how tissue microenvironment influence cell behavior and fate. Moreover, 3D cultures provide a more physiologically relevant culture system to test therapeutic efficacy [5,6]. In addition, there is significant progress in the application of biomaterials across the areas of biomedicine [7,8,9]. Hydrogels, intended for bioprinting, are important to downstream applications such as injectable drug delivery. Microfluidics are also rapidly emerging to develop innovative strategies in questions on stimuli-responsive, self-healing, composites, as well as other applications [10,11,12,13,14,15,16,17,18,19]. The area of biomaterials also include active studies to optimize and customize existing thermoplastic polymers like polycaprolactone [20]. Concurrently, there are development of new thermoplastic polymer backbones, such as polyester amides, pointing toward broader progress in the area of biomaterials [21]. Moreover, advancements of available biomaterials and fabrication technologies enable the creation of novel cell constructs. The cumulative result of the advancements in material science has led to success of 3D bioprinting to develop *de novo* tissues and organs with phenotype and function similar to their natural counterparts [22,23,24,25,26,27].

Bone marrow (BM) is a primary lymphoid organ and the site of hematopoiesis, which produced blood and immune cells throughout life. The BM is a complicated organ to replicate in vitro because it is a pliant, gelatinous tissue containing densely packed heterogeneous cells [28,29,30,31,32,33]. The development of biomimetic 3D in vitro BM models is critical for basic and applied research. First, hematological malignancies represent approximately 10% of new cancer diagnoses annually in the US, and based on current data, only an estimated 26% of the drugs for cancer will obtain FDA approval [2,34]. Thus, there is a need for BM models to predict drug responses. Second, there is a need to recapitulate the BM microenvironment to understand hematopoiesis during homeostasis and disease states. BM cell composition, biological properties, distribution, interaction with other cells and soluble and insoluble factors, and physical properties govern the fate of hematopoiesis [35,36].

The BM supports hematologic and non-hematologic malignancies. In some cancers, dormant cancer cells with the BM have been identified as a source of cancer relapse [37,38,39,40,41]. Moreover, toxicity of cancer drugs is generally identified by examining the balance between clinical benefit and harm to the endogenous hematopoietic stem cells, which at times, are reflected in improved overall survival, quality of life, and/or physical functioning [42,43,44].

In order to unlock the vast capabilities of 3D bioprinting for in vitro BM model development, it is imperative to design a hydrogel-based bioink amenable to extrusion that also sufficiently recapitulates the microenvironment. Although hydrogels are often used to mimic soft tissues, it is challenging to bioprint self-supporting mechanically stable hydrogels with an elastic modulus below 1 kPa [45,46]. Technical considerations related to mechanical and functional properties of hydrogels, including but not limited to injectability and permeability, can be critical to the development of bioinks as well as hydrogels for clinical injectables and other applications [47,48,49]. Shear thinning and recovery allow materials to be injected to an in vivo or in vitro destination and restore their structure, enabling improved retention of encapsulated factors (e.g., drugs, signaling molecules, cells) upon injection. In addition, these factors preserve porosity of the deposited structure, preserving permeability of the construct [50,51]. Moreover, experimental assessment of the aforementioned factors continues to be hindered due to a lack of standardized methods to characterize the optimal mechanical and biological bioink properties.

Informed by our experience with hematopoietic cell culture and bioinks previously reported in the literature, we created a bioink comprised of methylcellulose (MC) and alginate. MC is a thermally gelling polymer widely used as a binder, thickener, or emulsifier in industrial applications, but is gaining popularity for its value in biomedical applications, including drug delivery and cell culture [52,53,54,55,56]. Alginate, similarly, is a frequently used material for bioink development due to its rapid ion dependent crosslinking and shear thinning behavior [57,58].

In this study, MC-alginate hydrogel bioinks were fabricated according to the well-established use of MC matrices to study hematopoietic progenitors in colony forming assays. After in-depth characterization and evaluation, we determined two of the initial nine hydrogel formulations demonstrated properties aligned with both extrusion bioprinting and BM modeling: 4% (*w*/*v*) MC and 2% (*w*/*v*) alginate (4:2), and 6% (*w*/*v*) MC and 2% alginate (6:2). We purport that 4:2 bioink, in particular, more closely replicates select properties of BM than 6:2 bioink, such as gross architecture, availability of oxygen, and rheological characteristics. The bioinks reported in this study may serve as a foundational tool to model different BM microenvironment through desired cellular printing and also by tagging specific molecules to the scaffold that could facilitate cell adhesion, and release of soluble factors. We anticipate that 4:2 bioink will serve as a conduit between 2D in vitro and pre-clinical animal models to enhance the efficacy of translating the science and to improve on drug screening. These fundamental improvements may serve the critical gap between 2D in vitro and pre-clinical animal models, consequently to impact quality of life and survival.

## 2. Materials and Methods

### 2.1. Ethics Statement

BM mesenchymal stem cells (MSCs) and endothelial cells (ECs) were expanded from BM aspirates of healthy subjects between 18 and 35 years (described below). The Institutional Review Board of Rutgers, Newark, NJ, approved the protocol (Pro0120100318 with annual renewal). All subjects provided informed consent.

### 2.2. Reagents, Antibodies

Alginic acid sodium salt (alginate), 4000 cPs methylcellulose (MC), and phosphate-buffered saline (PBS) without Ca^2+^ and Mg^2+^, Accutase cell dissociation reagent, alginate lyase (alginase), Ficoll Hypaque, ZnCl_2_, KI, and I_2_ were purchased from Sigma-Aldrich (St. Louis, MO, USA); Iscove’s Modified Eagle Medium (IMEM), Dulbecco’s Modified Eagle Medium (DMEM), PBS with Ca^2+^ and Mg^2+^, and tissue culture grade liquid 7.5% sodium bicarbonate, and 1X insulin–transferrin–selenium from Life Technologies (Grand Island, NY, USA). L-glutamine (LG), Penicillin-Streptomycin (PS), fetal bovine serum (FBS) from ThermoFisher Scientific (Waltham, MA, USA), EGM-2 Endothelial Growth Media was purchased from Lonza (Walkersville MD), Lox-1 Hypoxia Probe from Organogenix (Kanagawa Japan), G418 sulfate solution from Gemini Bio-Products (West Sacramento, CA, USA), extra-pure calcium chloride dihydrate crystals from EM Science (Gibbstown, NJ, USA), Hoechst-33342, rhodamine phalloidin, DAPI, C_12_ Resazurin, and SYTOX Green from Invitrogen (Eugene, OR, USA).

Murine monoclonal anti-CD31 (1/1000 dilution) and -von Willebrand Factor (vWF) (1/1000 dilution) were purchased from Agilent Dako (Santa Clara, CA, USA); HRP-conjugated goat anti-murine IgG (1/2000 dilution) from ThermoFisher Scientific; rabbit anti-human vinculin (1/1000 dilution) from Abcam (Cambridge, MA, USA). The following fluorochrome-conjugated murine monoclonal antibodies were purchased from BD Biosciences (San Jose, CA, USA): CD90-FITC, CD45-FITC, CD73-PE, CD44-APC and isotype IgG with the respective fluorochromes.

#### 2.2.1. Flow Cytometry

MSCs were washed with 1× PBS, followed by labeling with specific antibodies at 1/20 final dilution of the following: anti-CD45-FITC, -CD90-FITC, -CD73-PE and -CD44-APC. The cells were incubated for 30 min at room temperature in the dark. This was followed by washing with 1× PBS. The cells were immediately analyzed on the FACS Calibur (BD Biosciences).

#### 2.2.2. Western Blot Analyses

Whole cell extracts were isolated using a cell lysis buffer (50 mM Tris-HCl (pH 7.4), 100 mM NaCl, 2 mM MgCl_2_, 10% glycerol, and 1% NP-40). The extracts (20 µg) were electrophoresed on 6% and 8% SDS-PAGE gels, prior probing against vWF and CD31, respectively. Proteins were transferred onto polyvinylidene difluoride membranes (Perkin Elmer, Boston, MA, USA). The membranes were incubated for 48 h at 4 °C on a rocker with anti-Cx31 or anti-vWF (dilutions mentioned above) in 3% non-fat milk, dissolved in 1× PBS tween. Next, the membranes were incubated with HRP-tagged IgG in 3% milk, dissolved in 1× PBS-tween for 1h at RT. The membranes were developed chemiluminescence using SuperSignal West Femto Maximum Sensitivity Substrate.

### 2.3. Bioink Fabrication

Sterile (autoclaved) alginate was mixed to homogeneity in 60 °C deionized water (dH_2_O). Following this, we slowly added sterile (autoclaved) MC with continuous mixing. The suspension was equilibrated to room temperature with chilled dH_2_O, followed by adding IMEM and sodium bicarbonate. After mixing to homogeneity at room temperature, the flask was transferred to 4 °C for 1 h to facilitate gelation. Bioinks were aliquoted and stored at 4 °C or −20 °C for short- or long-term storage, respectively. Prior to printing, the active bioink aliquots were brought to room temperature.

### 2.4. Rheological Analyses

Rheological tests were performed with a DHR-2 Rheometer (TA Instruments, New Castle, DE, USA). Bioinks were added to 40-mm parallel Peltier plate geometry (gap width: 1000 µm). Strain sweep (0.1–100% strain, at 2 Hz) and shear recovery (0.1% strain for 1 min followed by 4 repetitions of 100% strain for 1 min and 0.1% strain for 5 min) tests were performed at room temperature. 

### 2.5. Fidelity

The Rutgers University ‘R’ logos were printed on Parafilm and the constructs immediately imaged. Five distinct dimensions of constructs were measured using ImageJ (NIH, Bethesda, MD, USA) and compared to same dimensions of the CAD-designated geometry. The dimension differences were calculated by dividing the dimension of the printed construct by the CAD-designated dimension (printed/CAD).

### 2.6. Bioprinting

All model creation and bioprinting was performed using CELLINK^®^ INKREDIBLE 3D Bioprinter (CELLINK AB, Sweden). A 22G polypropylene nozzle (CELLINK AB, Sweden) was used to print all structures. Cell-free bioink (for cell-laden bioink, refer to “Preparation of cell-laden scaffolds” section below) was loaded into the printer cartridge with attached nozzle. Then, bioprinter X-Y-Z axes were calibrated, printing pressure was adjusted to ensure continuous flow, and scaffolds were printed. Assays described in the following sections used four-layer lattice cubic scaffolds (6-mm × 6-mm × 1.2-mm) in 24-well plates (TPP, Switzerland) unless otherwise noted. After bioprinting, scaffolds were crosslinked with sterile 100 mM CaCl_2_ solution for 5–10 min, washed with the appropriate culture solution for 20 min to remove excess CaCl_2_ and incubated in standard culture conditions (37 °C, 5% CO_2_, and 95% relative humidity).

### 2.7. BM Aspirates

BM mononuclear cells (MNCs) were isolated from BM aspirates from posterior iliac crests of healthy subjects between 18 and 35 years (see ethics statement above).

### 2.8. MSC Culture

MSCs were cultured from BM aspirate as described [59,60]. Briefly, unfractionated BM aspirates were diluted in DMEM containing 10% FBS and then seeded in plasma-treated Falcon 3003 Petri dishes. The plates were incubated at 37 °C, 5% CO_2_, and 95% relative humidity. After 72 h, the red blood cells and granulocytes were removed by Ficoll Hypaque density gradient and the MNCs replaced in the plates. At weekly intervals, 50% of the media were replaced with fresh media. At 80% confluence, the adherent cells were passaged. After passage 3, the cells were CD45^−^, CD44^+^, CD29^+^, CD90^+^, and CD73^+^. Each batch of MSCs showed multilineage differentiation into osteogenic and adipogenic cells.

### 2.9. EC Culture

MNCs from unfractionated BM aspirates (see above for isolation) were seeded in T25 tissue culture flasks (Greiner Bio-One, Monroe NC) at 10^7^/7 mL of EGM-2 Endothelial Growth Media. Confluence was noted at day 21. At this time, the adherent cells were serially passaged.

### 2.10. Preparation of Cell-Laden Scaffolds

Cells were dissociated with Accutase and then resuspended in 150 µL of media for mixing with 4:2 or 6:2 bioink. Mixing occurred with two Luer lock adapter-connected 3-mL syringes, resulting in 10^6^ cells/mL bioink. Cell-laden bioinks were loaded into cartridges, printed, and crosslinked with sterile 100 mM CaCl_2_ for 5–10 min. Scaffolds were then washed for 20 min in the appropriate culture solution then incubated in standard culture conditions (37 °C, 5% CO_2_, and 95% relative humidity).

### 2.11. Cell Viability and Vitality

LIVE/DEAD Cell Vitality Assay Kit (Invitrogen) assessed cell health, according to manufacturer instructions. Healthy living cells were stained red (C_12_ Resazurin), injured living cells, yellow or orange (C_12_ Resazurin + SYTOX Green), and dead cells, green (SYTOX Green). We acquired images on the Fluoview FV10i Confocal Microscope (Olympus, Tokyo Japan). Each time point included images from 8 technical and 4 biological replicates. The images were analyzed with FV10-ASW Viewer Software (Olympus) and ImageJ.

### 2.12. Cell Morphology

Scaffolds were fixed with a solution containing 90% methanol and 10% acetic acid for at least 20 min. Fixed scaffolds were washed twice with PBS without Ca^2+^ and Mg^2+^. Scaffolds containing MSCs and ECs were labeled with DAPI and rhodamine phalloidin. Images were acquired using a Fluoview FV10i Confocal Microscope. Images were analyzed using FV10-ASW Viewer Software and ImageJ.

### 2.13. Scanning Electron Microscopy

Prior to processing for scanning electron microscopy (SEM), printed cell-free scaffolds (4:2 and 6:2) were crosslinked, and cultured for 72 h in 10% FBS DMEM containing 1% (*v*/*v*) LG and 1% (*v*/*v*) PS. Scaffolds were fixed with glutaraldehyde, dehydrated via exchange with ethanol, and critical point dried. Dehydrated scaffolds were viewed using JSM-7900F Schottky Field Emission Scanning Electron Microscope (JEOL USA, Inc., Peabody, MA, USA) with an accelerating voltage of 2–3 kV.

### 2.14. Hypoxia Assessment

Bioink cell-laden scaffolds (4:2 and 6:2) were treated with 2 µM Lox-1 Hypoxia Probe at 48 h after printing. The scaffolds were incubated and after 24 h, imaged with the Fluoview FV10i Confocal Microscope. “Normoxic and hypoxic incubation” used standard culture conditions (37 °C, 5% CO_2_, 95% relative humidity). However, hypoxic conditions used parafilm sealed plates, which impeded gas exchange with the ambient environment. The presence or absence of red fluorescence of the Lox-1 probe was quantified with FV10-ASW Viewer Software and ImageJ.

### 2.15. Scaffold Degradation Assessment

Cell-free scaffolds (4:2 and 6:2) were cultured in 1 of 6 culture solutions: DMEM containing 1% (*v*/*v*) LG, 1% (*v*/*v*) PS, and either 10%, 2%, or 0% FBS, PBS with and without Ca^2+^ and Mg^2+^, and ultrapure dH_2_O. DMEM with 0% FBS was supplemented with 1X insulin–transferrin–selenium as for printing of cell-laden scaffolds. Scaffolds were photographed immediately following crosslinking (Week 0) followed by weekly intervals up to 12 weeks for observable degradation (e.g., loss of shape, fractionation). At the same weekly intervals, culture solutions were collected to measure free MC and Ca^2+^ as indicators of long-term MC elution and Ca^2+^-mediated alginate crosslink breakage, respectively, as described.

### 2.16. Methylcellulose Release Analyses

Cell-free scaffolds (4:2 and 6:2) were printed and cultured in the 6 aforementioned culture solutions. Culture solutions were collected weekly for 12 wks. All collected samples were stored in 1.5 mL centrifuge tubes at 4 °C until time of assay. A colorimetric assay was developed to quantify MC concentration in each solution. Media (150 µL) were added to the wells of 96-well plates. Chlorine-zinc-iodine (Cl-Zn-I) solution (50 µL) (see below for preparation) was added to each well followed by gently agitation for 5–10 min [61]. Absorbance was measured using a microplate reader at 500 nm [62]. MC concentration was calculated using a standard curve of 0.001 to 1% (*w*/*v*) aqueous MC. The MC concentrations are reported in µg/µL.

Cl-Zn-I solution followed a previously described protocol as follows [61]: 50 g of ZnCl_2_ was thoroughly mixed in 25 mL dH_2_O; 5 g KI and 0.25 g I_2_ were added to 125 mL dH_2_O and thoroughly mixed; the two solutions were combined and then stored in a glass bottle at room temperature.

### 2.17. Ca^2+^-Mediated Alginate Crosslink Breakage Analyses

Cell-free scaffolds (4:2 and 6:2) were printed and cultured in 1 of the 6 aforementioned culture solutions. The solutions were collected from the cultures at weekly intervals up to week 12. The samples were stored in 1.5 mL centrifuge tubes at 4 °C until ready for analyses. Ca^2+^ concentration was determined with a Calcium Colorimetric Assay Kit (Sigma-Aldrich, St. Louis, MO, USA) according to manufacturer instructions. Briefly, 50 µL of each sample was added to wells of 96-well plates with Chromogenic Reagent and Assay Buffer. Plates were gently agitated in the dark for 5–10 min. Absorbance was measured on a microplate reader at 570 and 600 nm and Ca^2+^ concentrations reported in µg/µL.

### 2.18. Scaffold Weight Fluctuation Measurements

Cell-free scaffolds (4:2 and 6:2) were printed and cultured in one of the six aforementioned culture solutions. The base line scaffold weight was determined immediately following crosslinking (W_0_) the evaluated for weights at 5, 30, and 90 min, and 1, 3, 7, 10, and 14 days post-printing (W_x_). Weight change ratio (W_x_/W_0_), indicating scaffold swelling or shrinkage, was calculated at each time point for each solution and compared to baseline (W_0_/W_0_).

### 2.19. Cell Line

MDA-MB-231 (highly invasive, basal-like) triple negative breast cancer cells (BCCs) were purchased from American Type Culture Collection (ATCC) and cultured per manufacturer’s instructions. The BCCs were stably transfected with pEGFP1-Oct3/4 as described [63]. Stable transfectants were maintained with G418 (50 mg/µL). The cells were authenticated with ATCC STR database (www.atcc.org/STR_database.aspx). Cells were also screened weekly for Mycoplasma.

### 2.20. Sorting of BCC Subsets

BCC subsets were selected as described [63]. Briefly, MDA-MB-231 with stable pOct4a-GFP were sorted on the FACSDiva fluorescence-assisted cell sorter (BD Biosciences, San Jose CA). The BCCs were sorted as per GFP intensity and the grouped into the following subsets: Oct4a-GFP^high^ (top 5% intensity), Oct4a-GFP^med^ (middle 20%), and Oct4a-GFP^low^ (bottom 5%), as described [64]. The Oct4a-GFP^high^ population contains mostly cancer stem cells (CSCs) [63].

### 2.21. Statistical Analysis

Student’s t-test compared differences between data points with Kruskal–Wallis post-hoc analysis unless otherwise stated. *P* values < 0.05 were considered statistically significant. Images from the cell health analyses were determine via 2-way ANOVA.

## 3. Results

### 3.1. Selection of Bioink Formulations

In order to recapitulate the BM microenvironment with 3D bioprinting, it is important to establish a bioink that maintains printability and stability. Thus, we created and characterized nine bioinks with varied concentrations of MC and/or alginate. We designated the bioink formulations based on the MC:alginate concentration (i.e., 1:2 = 1% [*w*/*v*] MC and 2% [*w*/*v*] alginate). Bioinks were scored and evaluated according to 4 objectively weighted criteria within a decision matrix: Ease of Fabrication (15%), Fidelity (20%), Handleability (30%), and Extrudability (35%) (Table S1). Results of the decision matrix identified two of the highest scoring nine bioinks for further study: 4% MC and 2% alginate (4:2) and 6% MC and 2% alginate (6:2).

### 3.2. Rheometric Analyses of Bioinks—Similarity to BM Tissue

**Figure 1:**
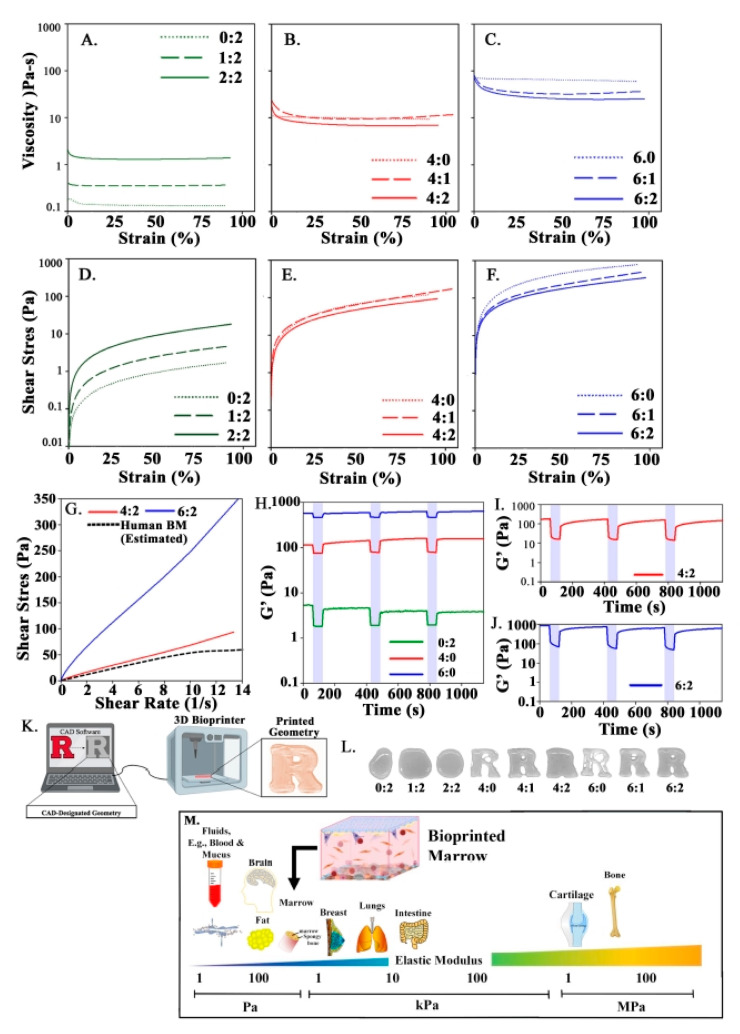
Rheological analyses of hydrogels. Strain sweeps were performed using oscillatory rheometry to measure viscosity (**A**–**C**) and flow (**D**–**F**) behaviors of hydrogels containing 0–2% (*w*/*v*) MC (green), 4% MC (red), and 6% MC (blue). (**G**) Flow curves for 4:2 (red) and 6:2 (blue) bioinks were compared to that of human BM from a 44 year old male reported by Sobotková [30]. Shear recovery analyses for (**H**) single component bioinks, (**I**) 4:2 bioink, and (**J**) 6:2 bioink. (**K**) Shape fidelity refers to the ability of a hydrogel to maintain the designated geometry once it is printed. (**L**) Assessment of shape fidelity of bioinks with printing of Rutgers University logo and assessing the gross degree to which the structures resembled the designated geometry. (**M**) Diagrammatic summary of the spectrum of elastic moduli (G’) of various bodily tissues and biomaterials as reported across the literature. Shown is femur, between 100 Pa and 100 kPa. The marrow is shown below 1 kPa, which is consistent with the perivascular and central niches.

Rheometric analyses to assess the mechanical behavior of the bioinks indicated that the concentration of MC was proportional to the viscosity and indirectly proportional to flow capacity (Figure 1A–F). We observed that increasing the MC concentration of the hydrogel by 2% (*w*/*v*) resulted in an order of magnitude increased viscosity and shear stress required for flow (Figure 1A–F). Additionally, bioinks exhibited shear-thinning behavior that closely correlated to the concentration of alginate in the hydrogel (Figure 1A–C). Shear thinning is advantageous for extrudability of bioinks and preservation of cell integrity, as well as mimicry of BM tissue architecture [30,31,65]. Importantly, rheological analyses showed similar flow properties to that of human BM tissue, reported by Sobotková and colleagues (Figure 1G).

The fidelity of bioprinted structures depends on the ability of a bioink to shear recover following extrusion. We observed an order of magnitude increase in viscosity per 2% increase in concentration of MC, and a similar increase in the shear modulus, or G’, of the hydrogels (Figure 1H–J). The G’ for 4:2 and 6:2 bioinks are comparable to bioinks of similar composition and fall within the range of those reported for BM tissue [30,32,66,67,68]. In shear recovery studies, the 4:2 and 6:2 bioinks were able to reach pre-shear G’ during the 5-min recovery period at low shear (0.01%). This behavior was also demonstrated with hydrogels of equivalent concentrations of MC or alginate (Figure 1H–J). However, full recovery of the 4:2 and 6:2 bioinks required a 5-min recovery period when they approached their pre-shear G’ (~9x% recovery). This contrasted with the result of equivalent single-component hydrogels, which recovered almost immediately (Figure 1H–J).

We corroborated shape fidelity by analyzing the rheological properties of hydrogels (Figure 1K). Bioinks with less or equal to 2% MC were unable to achieve or maintain the designated structure due to low viscosity and elastic modulus (Figure 1L). However, bioinks with 4% MC or 6% MC were more efficient at maintaining the designated structures (Figure 1L).

Although 4% MC bioinks were less viscous than 6% MC bioinks, the 4:0 bioink did not extrude well. Rather, the resulting constructs appeared as incomplete structures in some of the areas tested. In contrast, the 4:1 and 4:2 bioinks were extruded relatively easy due to their enriched shear thinning behavior, but still showed spreading that may be attributable to slow shear recovery. Altogether, the selected 4:2 and 6:2 bioinks (Table S1) showed rheological similarities to native BM tissue. Although, the 4:2 bioink was more representative of BM elastic modulus and flow properties.

We compared the mechanical properties of our bioprinted scaffolds with the literature on the elastic moduli (G’) of various bodily tissues and biomaterials (Figure 1M). Femur generally falls within a broad range between 100 Pa and 100 kPa. However, the marrow/inner material is below 1 kPa because the perivascular and central niches fall on the low end of this range. As noted in the diagram, the 3D structures with 4:2 MC-alginate bioink were similar to the marrow with respect to mechanical property.

### 3.3. Cell Health Following Extrusion

**Figure 2:**
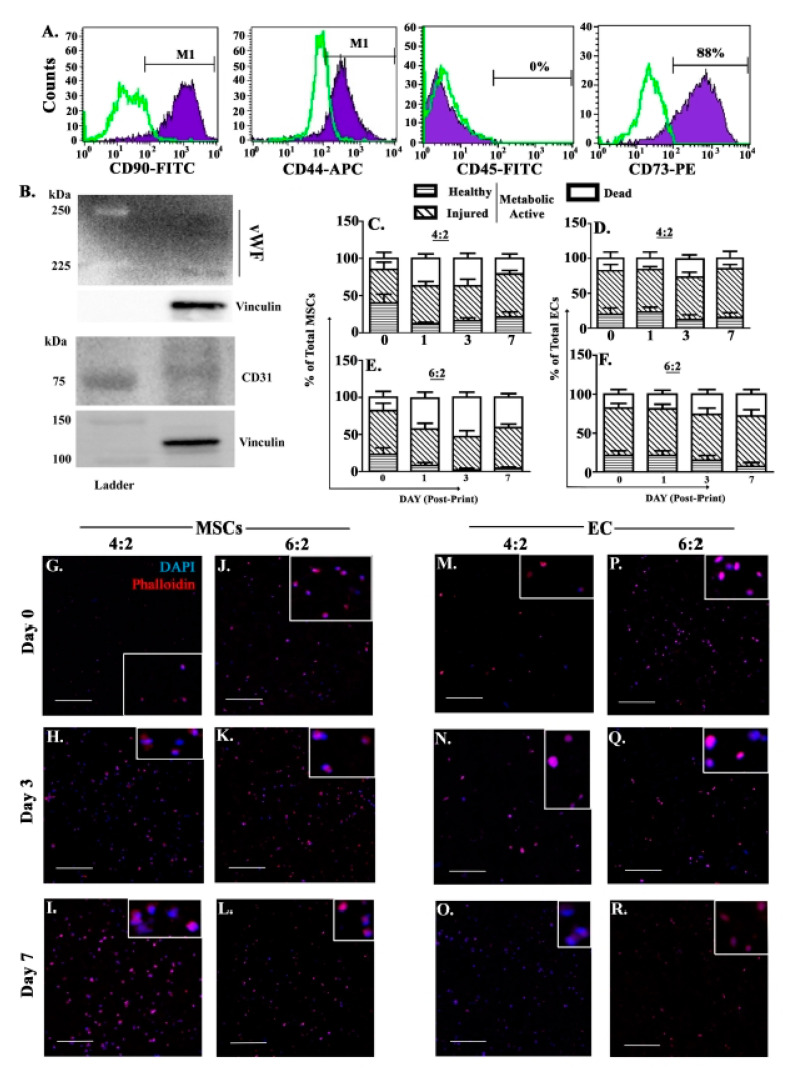
Cell viability and vitality are maintained by 4:2 and 6:2 bioinks. Flow cytometric analyses with MSCs labeled with anti-CD90, -44, -45 and -73 (**A**). Western blot with whole EC extracts for CD31 and vWF. Shown are the ladders that reflect the corresponding molecular weights of the test proteins (**B**). Primary human BM-derived MSCs and ECs were printed into 4:2 (**C**,**D**) and 6:2 (**E**,**F**) scaffolds, respectively. Timeline cell viability and vitality analyses were assessed up to day 7 as described in Materials and Methods. Viable cells are those deemed to be healthy and injured. The analyses represent 4 independent experiments, each with a different donor. Confocal microscopy imaged the cells at days 1, 3 and 7 within the scaffolds: MSCs in 4:2 (**G**–**I**) and 6:2 (**J**–**L**); ECs in 4:2 (**M**–**O**) and 6:2 (**P**–**R**). Shown are representative images at 10X magnification. Blue: DAPI (nuclei). Red: rhodamine phalloidin (actin). Scale bar: 250 µm.

The utility of 3D bioprinting is partly derived from the ability to print cell-embedded scaffolds [69]. Thus, it is crucial to evaluate the effect of extrusion on the health of printed cells by focusing on the selected 4:2 and 6:2 bioinks. We selected two primary human BM niche cells, MSCs and ECs since they are crucial cell types at the BM perivascular regions. The MSCs were cultured from human BM aspirates and by passage 3, they were free of hematopoietic cells as noted by negative labeling for CD45, and positive for CD90, CD73, CD44 and CD29 (Figure 2A). Western blots with whole cell extracts from human BM-derived ECs were positive for CD31 and vWF (Figure 2B)

The cells were printed using 25 kPa pressure into cubic scaffolds (dimensions: 6 mm × 6 mm × 1.2 mm) and evaluated after 7 days of culture using the Live/Dead cell vitality assay. Metabolically active cells were indicated when C12-resazurin transitioned into red-fluorescent C12-resorufin. The dead cells such as those in late apoptosis and necrotic uptake SYTOX^®^ Green dye, which demarcated metabolically active and dead cells.

Approximately half of the printed MSCs and ECs in both 4:2 and 6:2 bioinks showed reduced resorufin, indicating some form of membrane injury during extrusion (Figure 2C–F). However, since the presence of resorufin is an indication of metabolic activity, the observed fluorescence indicated that after one week in culture, >80% cultured cells recovered with metabolic activity.

MSCs printed in 6:2 bioink resulted in ~50% demonstrating metabolic activity, which is consistent with 6:2 bioink exhibiting relative increase in viscosity and reduced flow in the rheological studies (Figure 2E). Interestingly, printing of ECs showed metabolic activity similar to the 4:2 scaffolds (Figure 2C–F). Overall viability (metabolically active) for both MSCs in 4:2 bioink was approximately 80%, which is comparable to the viability achieved by bioinks with similar composition reported in the literature [70]. ECs showed better survival/metabolic activity in the 6:2 bioink as compared to MSCs.

### 3.4. Morphology of BM Miche Cells within Scaffolds

We developed the bioinks as a platform to recapitulate the BM perivascular region. This region is important, since it contains BM niche cells that facilitate cancer survival such as MSCs [71,72]. Since we were able to maintain cell health during the bioprinting, we evaluated the morphology of two perivascular cells, MSCs and ECs within the foundational 4:2 and 6:2 scaffolds during culture.

Timeline images using confocal microscopy revealed increased MSCs in the 4:2 scaffold at days 3 and 7 (Figure 2G–I). Although there was an increased number of cells in the 6:2 scaffold, the enhanced numbers were less pronounced, as compared to similar time points for the 4:2 scaffolds (Figure 2J–L). There was similar increase for EC in the 4:2 scaffolds (Figure 2M–O) but reduced cell numbers within the 6:2 scaffolds (Figure 2P–R). Furthermore, we noted little protrusion of actin filaments, based on evaluation of cells labeled with rhodamine phalloidin, suggesting tight adherence between the cells and the hydrogel structure (Figure 2E–P). In total, MSCs and ECs survived and proliferated within the 4:2 and 6:2 scaffolds.

### 3.5. Ultrastructure of Scaffold: Comparison with BM Structure

**Figure 3:**
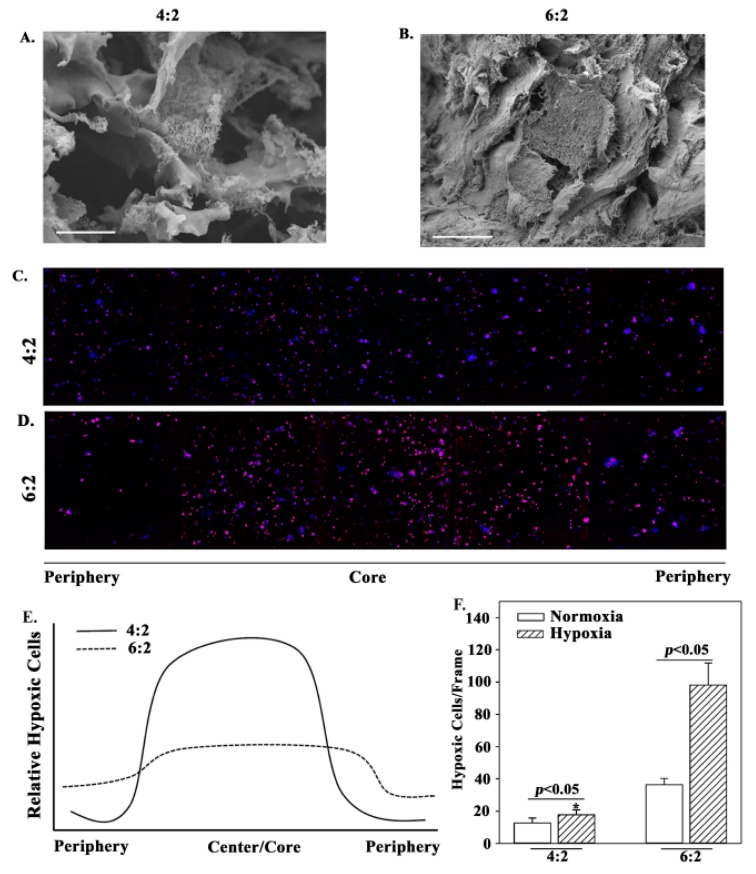
Scaffold ultrastructure and cell hypoxia. The ultrastructure of (**A**) 4:2 and (**B**) 6:2 cell-free scaffolds, by scanning electron microscopy (SEM). Shown are representative images at 2500X magnification. Scale bar: 10 µm. (**C**) Cells in 4:2 (**C**) and 6:2 (**D**) scaffolds were visualized by confocal microscopy for hypoxic cells from periphery to core. The number of hypoxic cells (expressing the Lox1 fluorescent hypoxia probe) in normoxic (open bar) or hypoxic (diagonal-stripped bar) incubation systems. Cells within (**C**) 4:2 and (**D**) 6:2 scaffolds treated with a Lox1 fluorescent hypoxia probe were imaged using confocal microscopy at 10X magnification. The relative differences between ‘C’ and ‘D’ along the structures are diagrammatically presented in (**E**). The number of normoxic and hypoxic cells were counted across 5 frames and the values presented as mean cells±SD (**F**), * *p* < 0.05 vs. 6:2 hypoxic cells. The images represent three different independent experiments.

The data in Figure 1 show similarities between the 4:2 bioinks and the mechanical properties of the marrow. Since we planned to study cancer cell behavior at the perivascular region and showed the survival of two major niche cells (Figure 2), we further analyzed the 4:2 and 6:2 bioinks for ultrastructural similarities with BM architecture. We subjected the scaffolds to scanning electron microscopy (SEM). At 72 h after printing, the images of both scaffolds revealed an ultrastructure comprised of a fibrous network with a large degree of heterogeneity (Figure 3A,B). However, the 4:2 scaffolds exhibited a more open structure with larger pores than the 6:2 scaffolds, which showed overall relatively condensed structure (Figure 3A,B). Furthermore, comparison of SEM images to previously reported images of human BM tissue uncovered striking similarities between 4:2 scaffolds and BM in terms of gross architecture, suggesting that 4:2 bioink may more closely mimic the structural organization of native BM [73,74].

The oxygen content varies within the BM cavity [75]. We therefore investigated how the oxygen availability within the scaffold ultrastructure affected the cells embedded in 4:2 and 6:2 scaffolds. To address this question, we used the Lox1 fluorescent hypoxia probe. Image analyses identified cells in hypoxic conditions in both scaffolds, although there was ~50% less hypoxic cells in the 4:2 scaffolds as compared to the 6:2 scaffolds (Figure 3C,D). Interestingly, we noted a hypoxic region in the core area of the 4:2 scaffold, which contrasted in the 6:2 scaffold with a relatively homogenous curve (Figure 3E). These observations were validated by quantitative analyses with significantly (*p* < 0.05) more hypoxic cells in the 6:2 scaffolds as compared to 4:2 scaffolds (Figure 3F). These results are consistent with the different pore size shown in the SEM analyses (Figure 3A,B). Together, the data indicate varied oxygen levels between 4:2 and 6:2 scaffolds with the latter showed continuous hypoxia across the structure.

### 3.6. Timeline Stability of Cultured Scaffold

Functional studies may require long-term cultures, e.g., studies to assess hematopoietic stem cells require > 6 wk cultures [76]. Therefore, to gauge the utility of 4:2 and 6:2 scaffolds for models associated with bone marrow functions, it was important to conduct analyses evaluating the stability of these scaffolds for long-term cultures. We cultured the scaffolds in DMEM with varying concentrations of FBS (0%, 2%, and 10%). We selected DMEM because it is widely utilized cell culture media for primary cell cultures. Control cultures contained deionized water or PBS, with or without Ca^2+^ and Mg^2+^.

**Figure 4:**
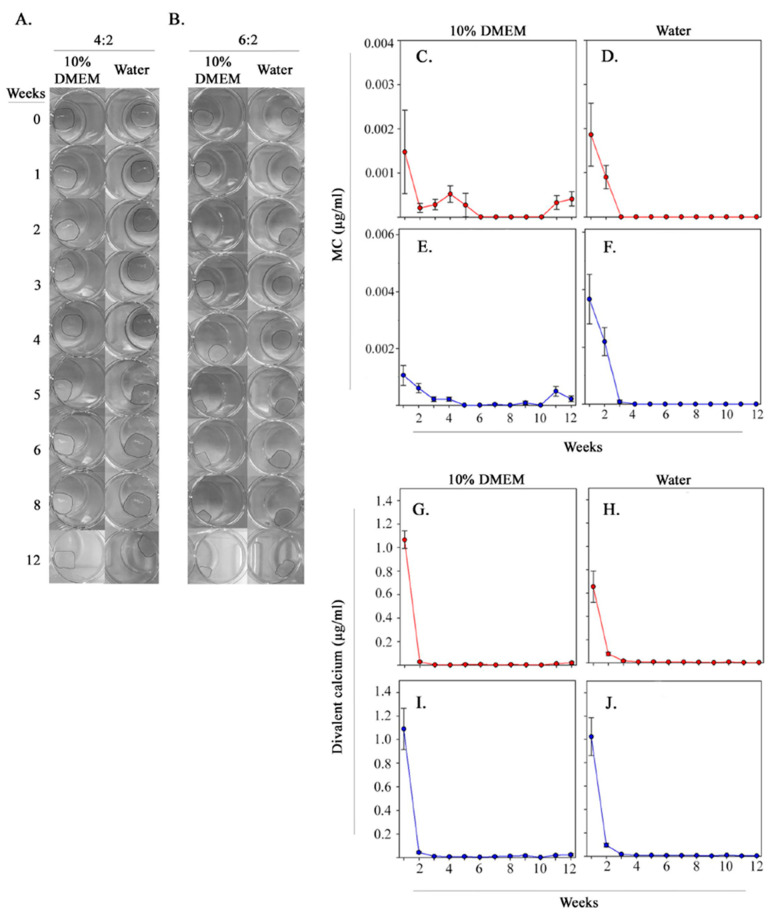
Stability of scaffold components in long-term culture conditions. Photographic images of (**A**) 4:2 and (**B**) 6:2 scaffolds were taken weekly for 12 weeks in 10% DMEM and deionized water, showing little bulk degradation of scaffold structure. MC (**C**–**F**) and calcium (**G**–**J**) concentrations in solution were measured weekly over 12 weeks for both 4:2 and 6:2 scaffolds to assess crosslink breakage over the culture period.

Weekly images of the 4:2 and 6:2 scaffolds revealed that scaffolds remained largely intact over 12 weeks (Figure 4A,B, Figures S1 and S2). However, MC and alginate crosslinks may degrade over time due to physical and ionic nature, respectively [61,77,78,79]. We therefore sought to monitor the stability of these scaffold components during the culture. To achieve this, we assessed weekly concentrations of MC and Ca^2+^ (from Ca^2+^-mediated alginate crosslinks) in the culture solution (Figure 4C–J and Figures S3–S6). The highest concentrations of MC and Ca^2+^ from 4:2 and 6:2 scaffolds were consistently measured in the first 2 weeks of culture with 10% DMEM and dH_2_O (Figure 4C–J). After this period, the concentrations decreased and remained at undetectable levels throughout the remainder the culture period. However, there was slight fluctuation in MC release when the 4:2 and 6:2 scaffolds were placed in 10% DMEM relative to increased release in deionized water (Figure 4C–J). The relatively high levels of MC and alginate crosslink breakage in the 4:2 and 6:2 scaffolds during the first 2 weeks did not significantly affect scaffold integrity based on weight changes, which did not exceed ±20% initial weight (Figures S7 and S8). Altogether, the data indicated stability of 4:2 and 6:2 scaffolds in common culture conditions, reinforcing their utility for long-term BM modeling applications.

### 3.7. Breast Cancer (BC) Cell Behavior in the 3D Scaffold

The data thus far indicated that the 4:2 bioink more closely reflects the properties of native BM tissue as compared to the 6:2 bioink. Since BC cells show preference for the BM [37,38,59,63,71,80,81,82,83,84,85], we sought to investigate how BC cells behave in the 4:2 scaffolds. One mechanism through which BC cells evade treatment and survive for long periods in the BM is by entering into dormancy. Once of the hallmarks of identifying dormancy is increased expression of stem cell genes [63]. Therefore, we sought to determine whether such transition of BC cells could be recapitulated in the 3D bioprinted BM model.

MDA-MB-231 triple-negative BC cells with stable pOct4a-GFP was used to demarcate different subsets of cancer cells [63]. GFP intensity is a surrogate of *Oct4a* gene expression. We sorted the MDA-MB-231 based on GFP intensity and designated them as low, medium, and high Oct4a expression. We previously reported on the population with the highest GFP intensity contained drug resistant cancer stem cells that could become dormant [63].

**Figure 5:**
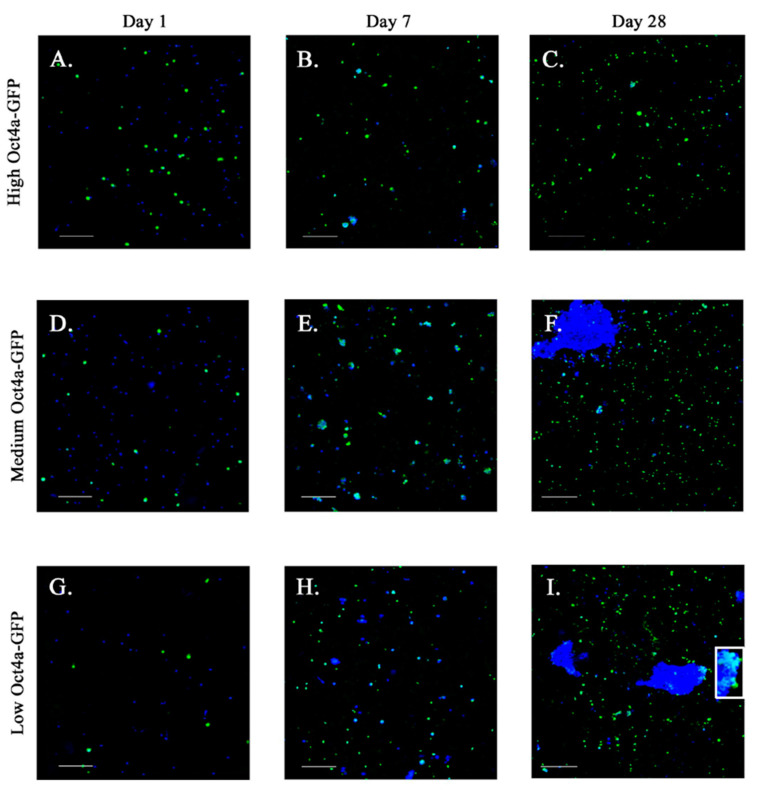
Behavior of bioprinted BC cell subsets in 4:2 scaffolds. MDA-MB-231 breast cancer cells (BCCs) were sorted (**A**–**C**) as Oct4a GFP-high (cancer stem cell, CSC), (**D**–**F**) GFP-medium, and (**G**–**I**) GFP-low cells. The three BCC subsets were printed into 4:2 scaffolds. Confocal images of fixed scaffolds were acquired at days 1 (**A**,**D**,**G**), 7 (**B**,**E**,**H**), and 28 (**C**,**F**,**I**). Representative images at 10× magnification are shown (n = 3 with 4 technical replicates). Blue: DAPI (nuclei). Green: GFP (Oct4a). Scale bar: 250 µm.

We performed a timeline imaging of the cells for GFP in culture. After 7 days, 4:2 scaffolds seeded with high and medium GFP intensities showed little change with respect to the cell numbers (Figure 5A,B,D,E). However, the scaffolds seeded with low GFP showed an increase in cells with GFP by day 7 (Figure 5G,H). After 28 days, there were increases in GFP in all groups (Figure 5C,F,I). More importantly, we noted clusters of BC cells at day 28 with initiating printing of non-cancer stem cells (Oct4a low and medium), shown in blue (DAPI) clusters. Since the initial plated cells were GFP-negative, the GFP cells at day 28 strongly suggested that the 3D scaffold induced cell dedifferentiation. Since no other BM cells were in the culture, this indicated cell autonomous method of dedifferentiation. In summary, by culturing metastatic cancer cells in BM biomimetic scaffolds, we were able to enrich the cancer stem cell-like cells, replicating an important aspect of cancer dormancy in BM.

## 4. Discussion

3D bioprinting has revolutionized the creation of 3D in vitro culture systems, allowing for rapid fabrication of constructs with pre-defined geometries. However, despite its success in creating a wide array of tissues in silico (e.g., breast, brain, lung, bladder), faithful replication of very soft tissues with 3D bioprinting has proven difficult. This is partly due to limited printability and low fidelity [86,87,88,89,90,91,92,93,94,95,96]. This is a significant obstacle because the degree of control over the composition, size, and structure of 3D bioprinted scaffolds can meaningfully influence the physiological and pathological models, as well as screening for drug performance. In light of this, it is not surprising that relatively little has been reported regarding biomaterials designed to specifically recapitulate the distinct physiochemical and cellular characteristics of BM, a semi-solid tissue with an elastic modulus as low as 250 Pa and a viscosity ranging from 0.04 and 125 Pa-s [30,32,67,68,97,98].

MC-alginate composite hydrogels are emerging as effective in formulating bioink for extrusion bioprinting applications. Their composition have the ability to control shape fidelity and shear thinning when incorporated in bioinks [70]. In designing the composition reported in this report, we incorporated technical outcome with respect to mechanical and functional properties of hydrogels with injectability [47,48,49]. Thus, we report on the method used to develop a scaffold with MC and alginate. The bioink printed structures that recapitulate the BM microenvironment, in particularly, structures resembling the perivascular region, which interfaces the periphery and marrow cavity. We selected MC due to its success in maintaining hematopoietic cells and then added alginate to retain the structure with 3D bioprinting.

Among the formulations studied, we selected 4% (*w*/*v*) MC and 2% (*w*/*v*) alginate (4:2) due to close similarity with BM structure (Figure 1G and Figure 3). In addition, these two formulations, in particular the 4:2, maintained primary human MSCs and ECs (Figure 2). Interestingly, the 4:2 structure showed a curved pattern of hypoxia, which closely mimics the BM cavity [99].

The following observations led to our final selection of 4:2 MC:alginate scaffold for future application to study BM structures in 3D models: (i) the bioink mimics the rheological and gross ultrastructural characteristics of BM (Figure 1 and Figure 3); (ii) the bioink shows no significant negative impact cell viability and vitality, and (iii) the scaffolds maintain stability in long-term culture. Furthermore, although the shape fidelity achieved using 4:2 bioink alone may not be suitable for creating large-scale or complex geometries, printing 4:2 bioink alongside a more rigid acellular “support” bioink would likely improve shape fidelity and facilitate the development of increasingly intricate structures.

The studies within the scaffold provided interesting findings. We did not attach adhesion molecules to our bioink. Yet, the behavior of breast cancer cells within scaffolds indicate that they are good models to study cancer metastasis. We observed clustered breast cancer cells over time in the scaffold (Figure 5). The Oct4a-hi population, which are mostly cancer stem cells [63] remained as single cells at Day 28 but retained stemness based on bright GFP (Figure 5C). This pattern was consistent with Oct4a-hi breast cancer cells being able to adapt as single cell dormancy in the BM [63]. Although the seeded Oct4a-med and –lo populations were dim for GFP, over time, they gained brighter GFP and formed cell clusters, which is consistent with metastatic lesion (Figure 5F,I). Since the cells showed no evidence of spreading, increased cell numbers as clusters suggested that the cells might secrete extracellular matrix that provided them with a basement to survive (Figure 5).

We concluded that Oct4a-lo breast cancer cells, which began as cells with no evidence of stemness, dedifferentiate into immature functional cells. Indeed, we showed dedifferentiation of Oct4a-lo breast cancer cells when exposed to MSC exosomes. However, the 3D scaffold did not contain BM accessory cells, indicating that dedifferentiation must be cell autonomous. This was an interesting finding because when we used 2D cultures, we observed no evidence of cell autonomous dedifferentiation. These findings indicate the importance of the model to discern how cancer cells survive as drug resistant cells in the BM, and to study cell autonomous mechanism of BM metastasis. Thus, the described bioink is highly significant to move forward with investigations on breast cancer behavior in BM. This is particularly important since the BM is the preferred site for breast and other cancers [81,83,84,85,100,101,102].

We evaluated mechanical similarity between bioinks (4:2 and 6:2) and endogenous BM, based on widely ranging viscosity and modulus data in the literature [30,32,66,67,68]. Variations in reported measurements may be attributable to inconsistencies in experimental approaches, such as the use of different model systems (e.g., human, bovine, porcine), sample freshness and storage method, cellular composition of samples, temperature at the time of measurements, and rheometer setup. Indeed, viscosity decreases with both temperature and shear rate, and is dependent on skeletal site [32,66].

The described biomaterial and model are foundational and translatable with modified parameters according to the desired research application and question. For example, alginate is particularly well known in tissue engineering to exhibit poor adhesion since mammalian cells do not express receptors for alginate, and low protein adsorption to alginate gels [57]. However, alginate can serve as the backbone to integrate specific cell adhesion molecules, such as coupling of RGD sequences, to fabricate the bioink. Indeed, the results are in line with the extensive use of these materials for cell encapsulation applications [53,57,70]. We printed cells sparsely at a density of 10^6^ cells/mL bioink. Increased cell numbers in the bioink would facilitate intercellular interaction. Similarly, the bioink structures can incorporate supplements with soluble factors such as cytokines and insoluble factors, e.g., extracellular vesicles in cultures.

A major application for the bioink is to recapitulate the hematopoietic microenvironment with specific BM cells. Such models could be the platform in low-cost method to screen drugs. Additionally, the cell containing scaffolds could be a cost-effective rapid method to evaluate drug for cancer cells in different regions within BM. This type of model could be expanded to evaluate drugs for toxicity; specifically, effective targeting of cancer cells without untoward effects on hematopoietic stem cells. Indeed, we intentionally selected the MC base to allow healthy hematopoietic cells to survive.

We did not find an overt issue with hypoxia with respect to EC and MSC viability during the first week of culture. However, oxygen availability may be significant in long-term cultures. This could be overcome with customized bioactive features, as desired by the end user, e.g., adding cell adhesion sequences or introducing soluble signaling factors.

The characterized scaffolds require in-depth analyses of the cells after printing. This includes the current MSCs and ECs but with other cells types to closely recapitulate the BM microenvironment. Based on the data from this study, the primary BM cells will survive in the bioink for long-term studies. In this study, the printed MSCs increased with time, strongly suggesting retention of multipotency. Cell death or differentiation would prevent their proliferation. ECs did not adhere to the bioink. However, long-term cultures with EC alone will determine if these cells can deposit their own matrix to form angiogenic tubes.

We are in the process of determining of determining how MSC secretome affect dedifferentiation, which we observed in 2D system. We were surprised because in the absence of accessory BM cells, the breast cancer cells showed evidence of dedifferentiation. It is possible that in 3D, MSCs might enhance breast cancer dedifferentiation and the structures will allow us to dissect cell autonomous process from intercellular communication. The 2D culture system failed to provide such difference. Another issue is the need to study the migration of ECs from the periphery to the central part of the marrow.

## 5. Conclusions

The MC-alginate bioink reported in this study is a promising material for the development of future platforms for BM modeling. Addition of MC to low-concentration alginate enhanced printability, mimicked the rheological and ultrastructural properties of BM tissue, and supported cell viability and vitality during extrusion and subsequent cell culture. In addition, scaffolds were relatively stable and suitable for long-term cultures. Thus, this MC-alginate bioink offers a foundation for modification with bioactive factors and tissue-specific components to enhance BM mimicry. Furthermore, the expression of stem cell genes by cancer cells is a key method through which cancer cells use to survive in BM and resist treatment. We provide evidence to show that 4:2 scaffolds closely replicating the structure of BM. We also showed that the structure could be applied to study breast cancer behavior in BM. We anticipate that this material can serve as a model to study the healthy microenvironment of the BM, and to uncover new methods to target elusive cancer cells and improve clinical outcomes.

## Data Availability

The data presented in this study are available on request from the corresponding author.

## Supplementary Materials

The following are available online at https://www.mdpi.com/2073-4360/13/4/480/s1, Table S1: Decision matrix, Figure S1: Visible degradation of 4:2 scaffolds in culture solutions, Figure S2: Visible degradation of 6:2 scaffolds in culture solutions, Figure S3: MC released from 4:2 scaffolds during long-term culture, Figure S4: MC released from 6:2 scaffolds during long-term culture. Figure S5: Alginate crosslink breakage in 4:2 scaffolds during long-term culture. Figure S6: Alginate crosslink breakage in 6:2 scaffolds during long-term culture, Figure S7: Weight fluctuation of 4:2 scaffolds during the first two weeks of culture, Figure S8: Weight fluctuation of 6:2 scaffolds during the first two weeks of culture.
