# Supplementary material for: A 3D Bioprinted Material That Recapitulates the Perivascular Bone Marrow Structure for Sustained Hematopoietic and Cancer Models
Source: Polymers (Basel). 2021 Feb 3;13(4):480. doi: 10.3390/polym13040480 (PMC7913313; doi:10.3390/polym13040480)
Supplement: Supplementary file 1 [file polymers-13-00480-s001.pdf]

SUPPLEMENTAL INFORMATION

Title: 3D Bioprinted Material that Recapitulates the Perivascular Bone Marrow Structure for Sustained Hematopoietic and Cancer Models.

Authors: Caitlyn A. Moore [a,b], Zain Siddiqui [c], Griffin J. Carney [a], Yahaira Naaldijk [d], Khadidiatou Guiro [b], Alejandra Ferrer [a,b], Lauren S. Sherman [a,b], Murat Guvendiren, Vivek A. Kumar [c,e,f], Pranela Rameshwar [a,b*]

Bioink Name Designation:				0:2	1:2	2:2	4:0	4:1	4:2	6:0	6:1	6:2
Bioink Components (% w/v)			MC	0	1	2	4	4	4	6	6	6
			Alginate	2	2	2	0	1	2	0	1	2
Performance Criteria	Rank	Weight										
Extrudability	1	35%		0.00	0.35	0.35	0.35	0.70	1.05	0.35	0.35	0.70
Handleability	2	30%		0.00	0.00	0.00	0.00	0.30	0.90	0.00	0.30	0.90
Resolution and Fidelity	3	20%		0.00	0.00	0.00	0.20	0.40	0.40	0.20	0.40	0.40
Ease of Fabrication	4	15%		0.45	0.45	0.45	0.45	0.45	0.45	0.30	0.15	0.15
Overall Performance:				0.45	0.80	0.80	1.00	1.85	2.80	0.85	1.20	2.15

Table S1: A decision matrix was established to identify potential candidate methylcellulose (MC)-alginate bioink formulations. Bioinks were grouped based on low (**green**), medium (**red**), or high (**blue**) relative MC content, and compared using 4 arbitrarily-weighted performance criteria: extrudability, handleability, resolution and fidelity, and ease of fabrication. Performance of bioinks across these criteria was scored on a scale of 0 (**poor**) to 3 (**excellent**) and totaled to provide an overall performance score. Bioinks with the highest performance scores (**black arrows**) were considered to be most feasible for use and were selected for further study.

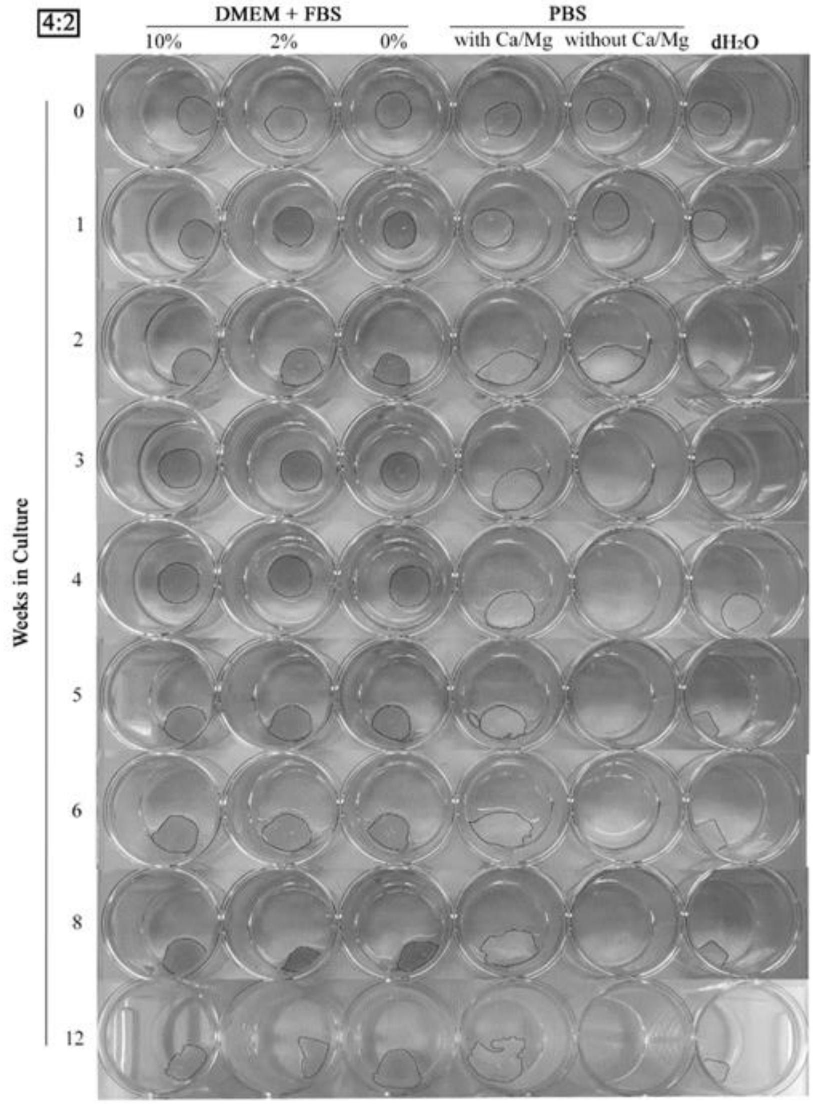

Figure S1. Visible degradation of 4:2 scaffolds in culture solutions. Images of 4:2 scaffolds were acquired weekly for 12 weeks (labeled left; weeks 7, 9, 10, 11 omitted). Scaffolds were cultured in DMEM with 10%, 2%, or 0% FBS, PBS with and without Ca^{2+} and Mg^{2+}, and deionized water (dH_2O) (labeled top).

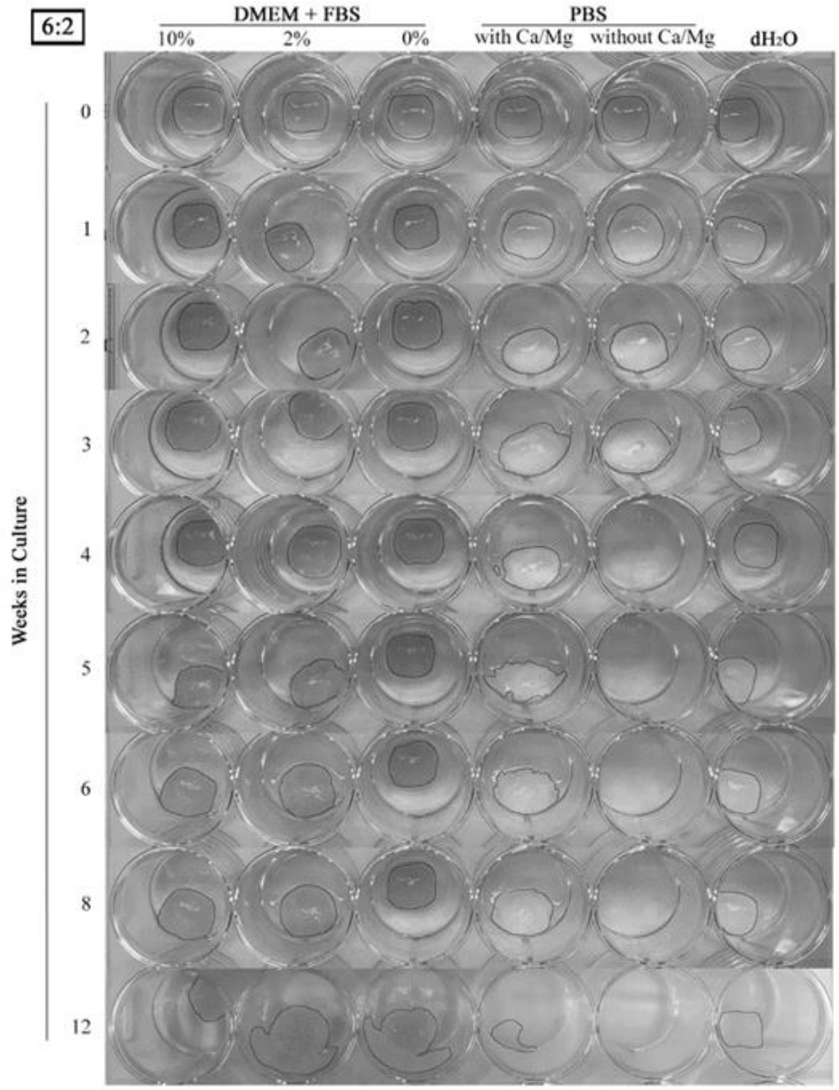

Figure S2. Visible degradation of 6:2 scaffolds in culture solutions. Images of 6:2 scaffolds were acquired weekly for 12 weeks (labeled left; weeks 7, 9, 10, 11 omitted). Scaffolds were cultured in DMEM with 10%, 2%, or 0% FBS, PBS with and without Ca^{2+} and Mg^{2+}, and deionized water (dH_2O) (labeled top).

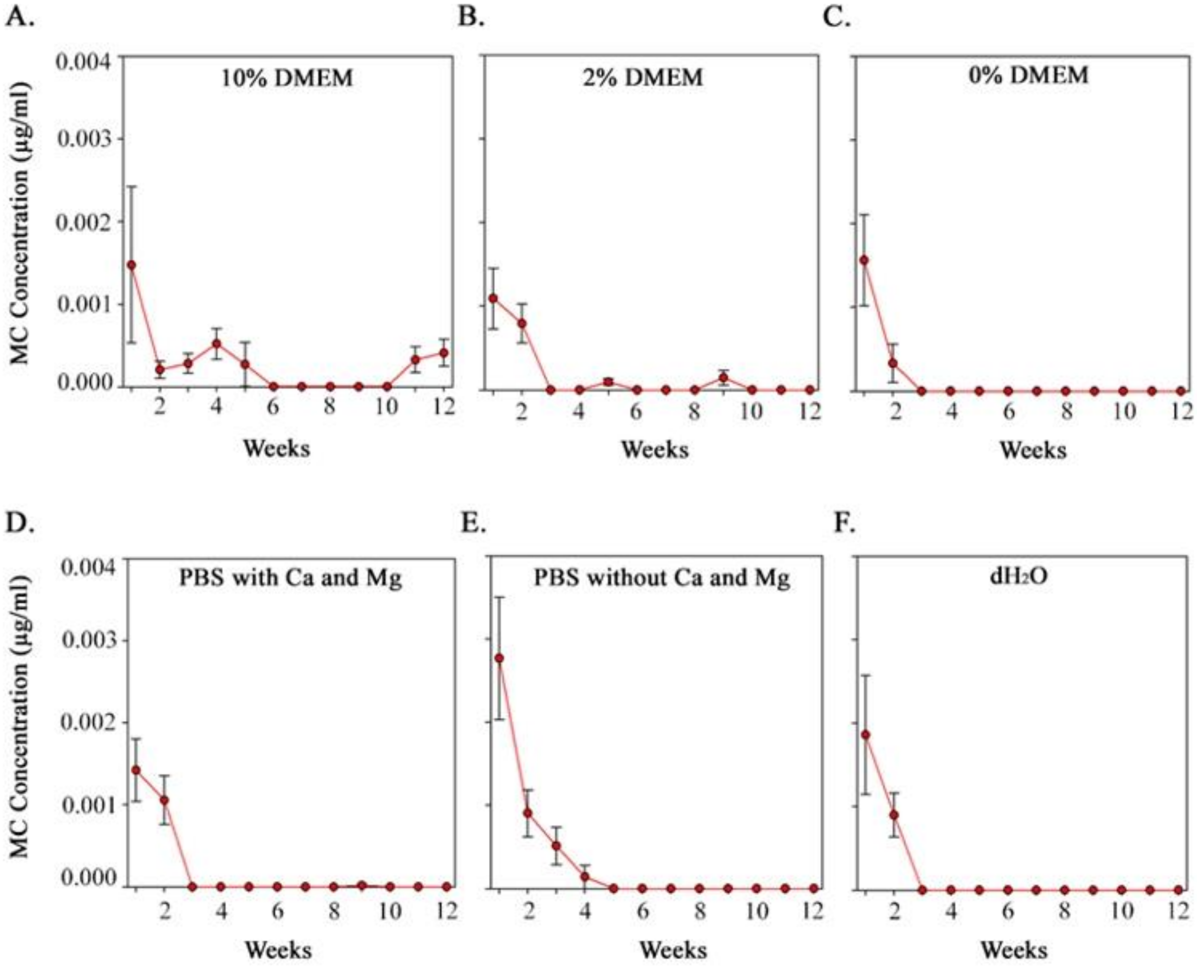

Figure S3. MC released from 4:2 scaffolds during long-term culture. The concentration of MC released from 4:2 scaffolds was measured weekly in culture solutions: DMEM containing (**A**) 10% FBS, (**B**) 2% FBS, or (**C**) 0% FBS, (**D**) PBS with Ca^{2+} and Mg^{2+}, (**E**) PBS without Ca^{2+} and Mg^{2+}, and (**F**) deionized water (dH_2O). Data represents n = 3 with at least four technical replicates.

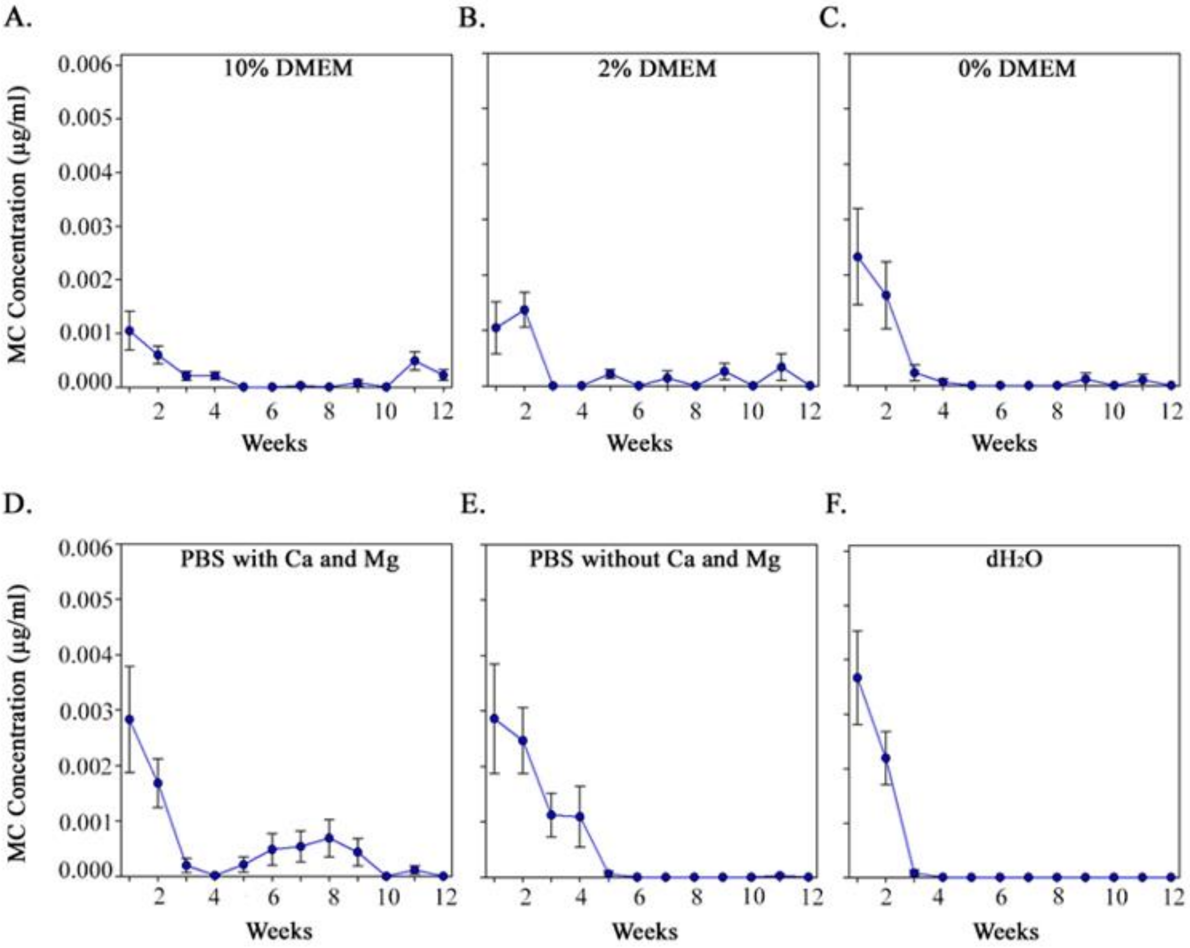

Figure S4. MC released from 6:2 scaffolds during long-term culture. The concentration of MC released from 4:2 scaffolds was measured weekly in culture solutions: DMEM containing (**A**) 10% FBS, (**B**) 2% FBS, or (**C**) 0% FBS, (**D**) PBS with Ca^{2+} and Mg^{2+}, (**E**) PBS without Ca^{2+} and Mg^{2+}, and (**F**) deionized water (dH_2O). Data represents n = 3 with at least four technical replicates.

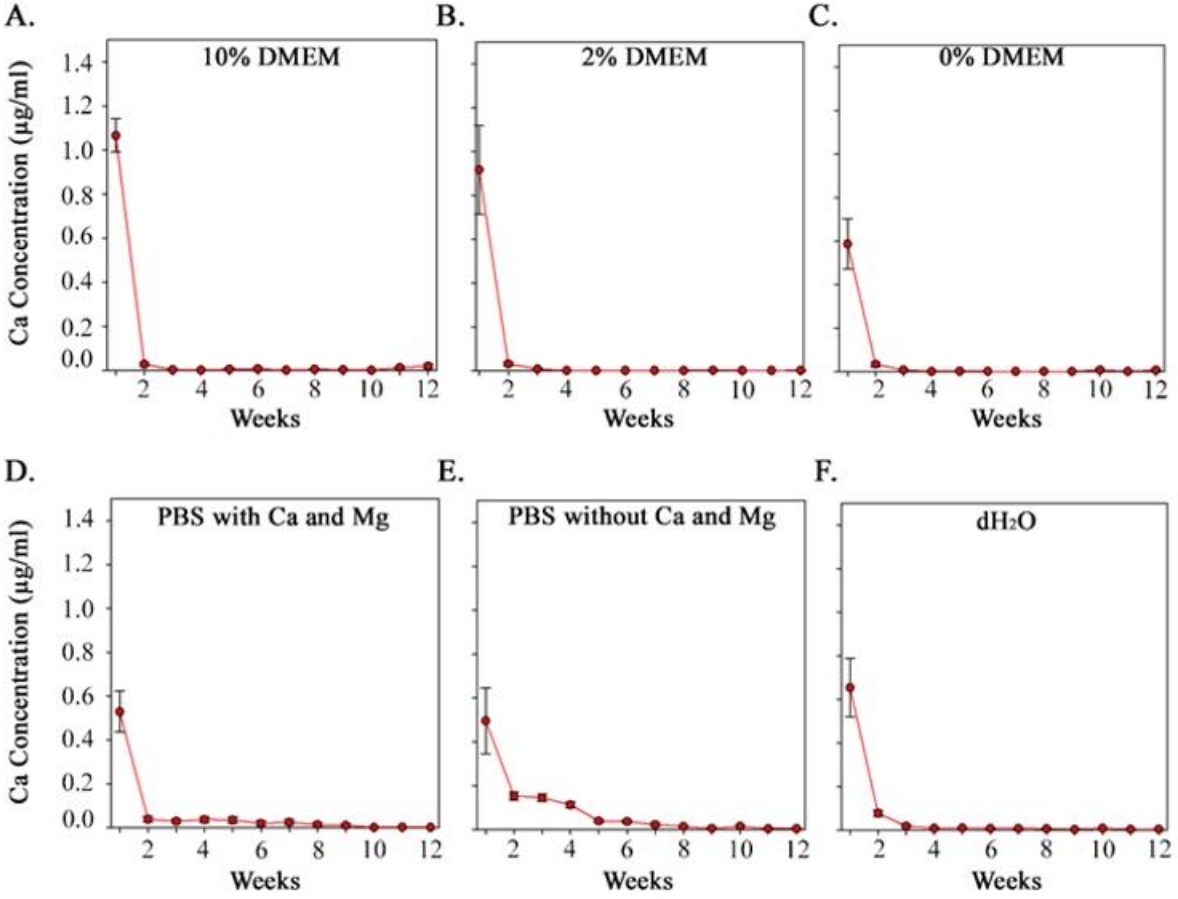

Figure S5. Alginate crosslink breakage in 4:2 scaffolds during long-term culture. Ca^{2+} in solution served as an indicator for alginate crosslink breakage in 4:2 scaffolds cultured in various solutions: DMEM containing (**A**) 10% FBS, (**B**) 2% FBS, or (**C**) 0% FBS, (**D**) PBS with Ca^{2+} and Mg^{2+}, (**E**) PBS without Ca^{2+} and Mg^{2+}, and (**F**) deionized water (dH_2O). Data represents n = 3 with at least four technical replicates.

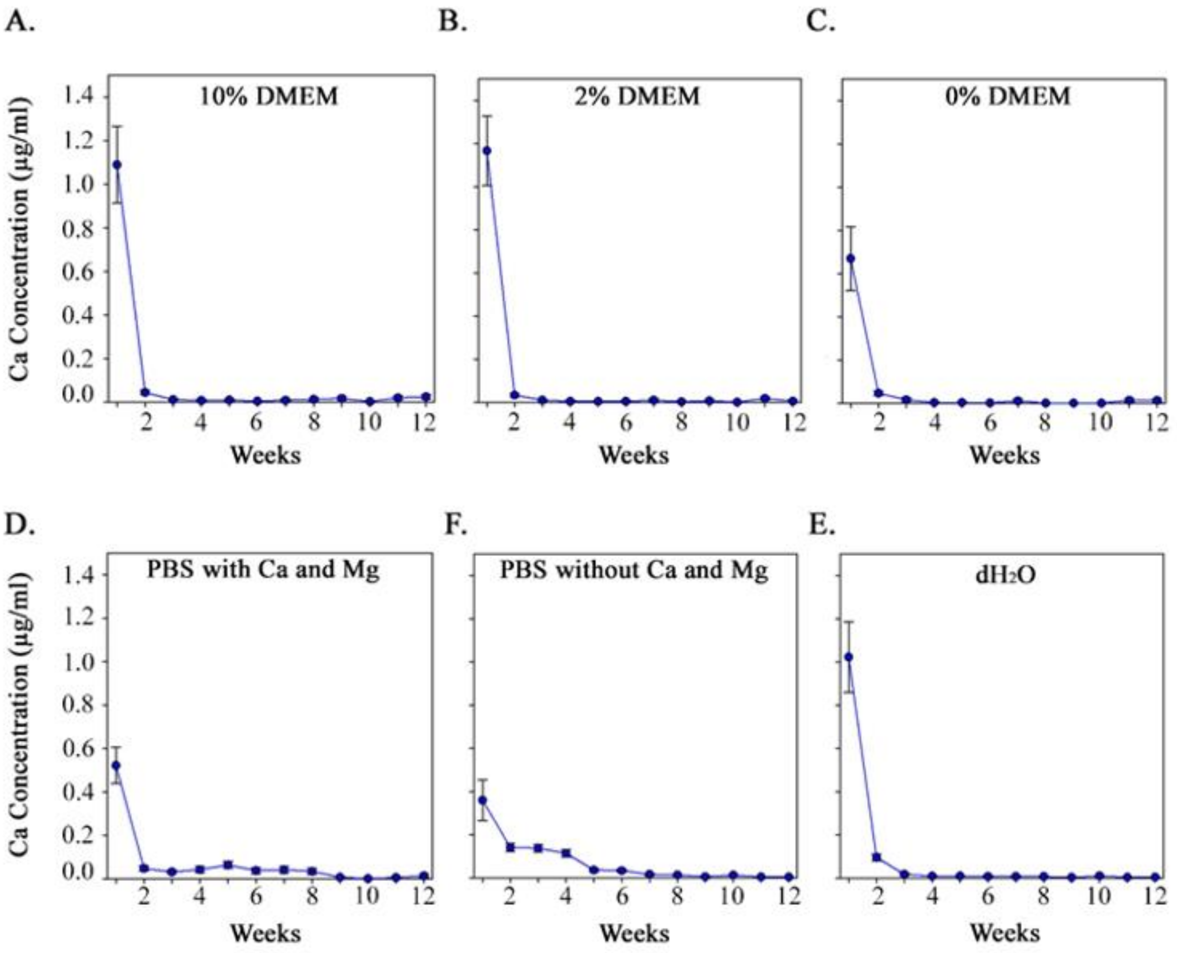

Figure S6. Alginate crosslink breakage in 6:2 scaffolds during long-term culture. Ca^{2+} in solution served as an indicator for alginate crosslink breakage in 4:2 scaffolds cultured in various solutions: DMEM containing (**A**) 10% FBS, (**B**) 2% FBS, or (**C**) 0% FBS, (**D**) PBS with Ca^{2+} and Mg^{2+}, (**E**) PBS without Ca^{2+} and Mg^{2+}, and (**F**) deionized water (dH_2O). Data represents n = 3 with at least four technical replicates.

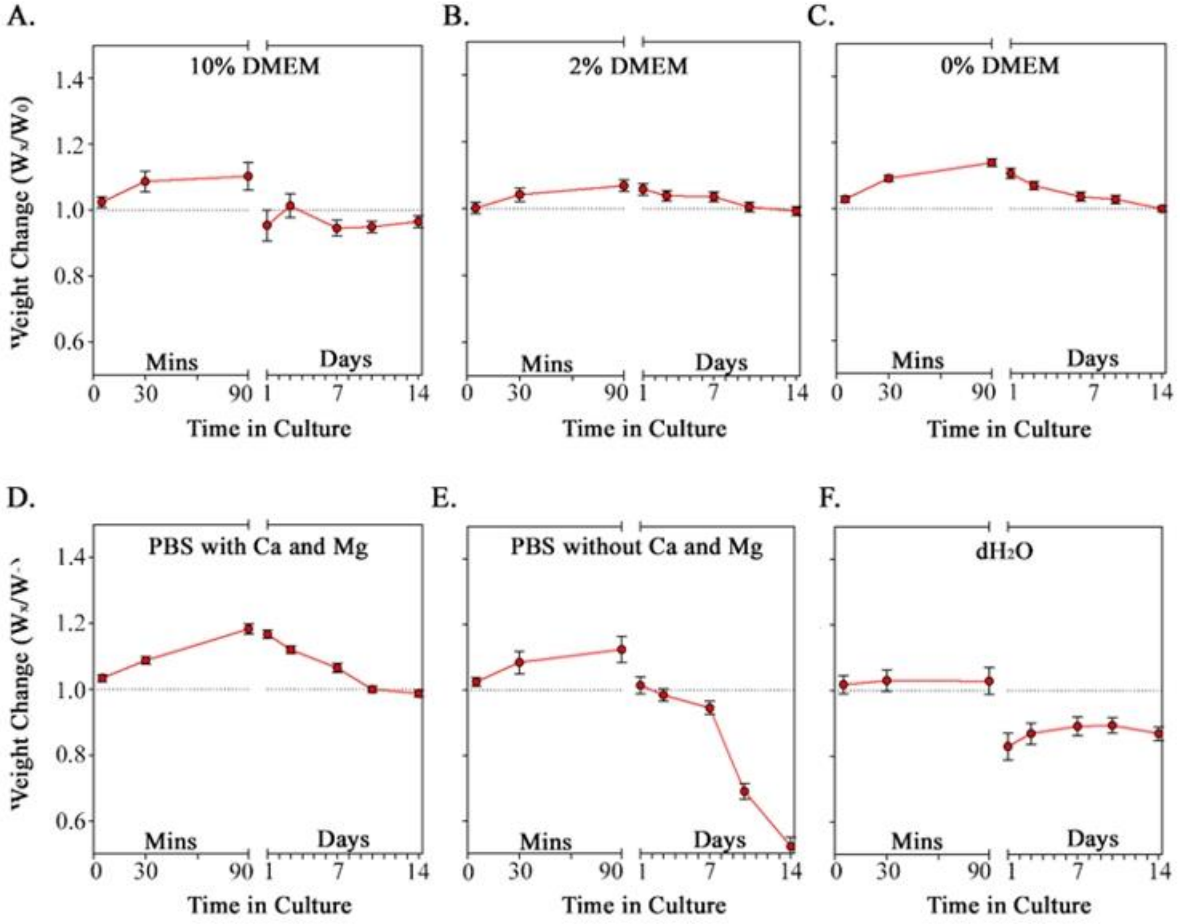

Figure S7. Weight fluctuation of 4:2 scaffolds during the first two weeks of culture. Weight of 4:2 scaffolds was measured at various times after printing (W_x) and normalized by initial weight (immediately after crosslinking, W_0) to yield a weight change ratio (W_x/W_0) for each scaffold. Scaffolds were cultured in Dulbecco's Modified Eagle Medium (DMEM) containing (**A**) 10%, (**B**) 2%, or (**C**) 0% fetal bovine serum (FBS), (**D**) PBS with Ca^{2+} and Mg^{2+}, (**E**) PBS without Ca^{2+} and Mg^{2+}, and (**F**) deionized water (dH_2O). Dotted line indicates initial weight (W_0/W_0). Data represents n = 4 with at least three technical replicates.

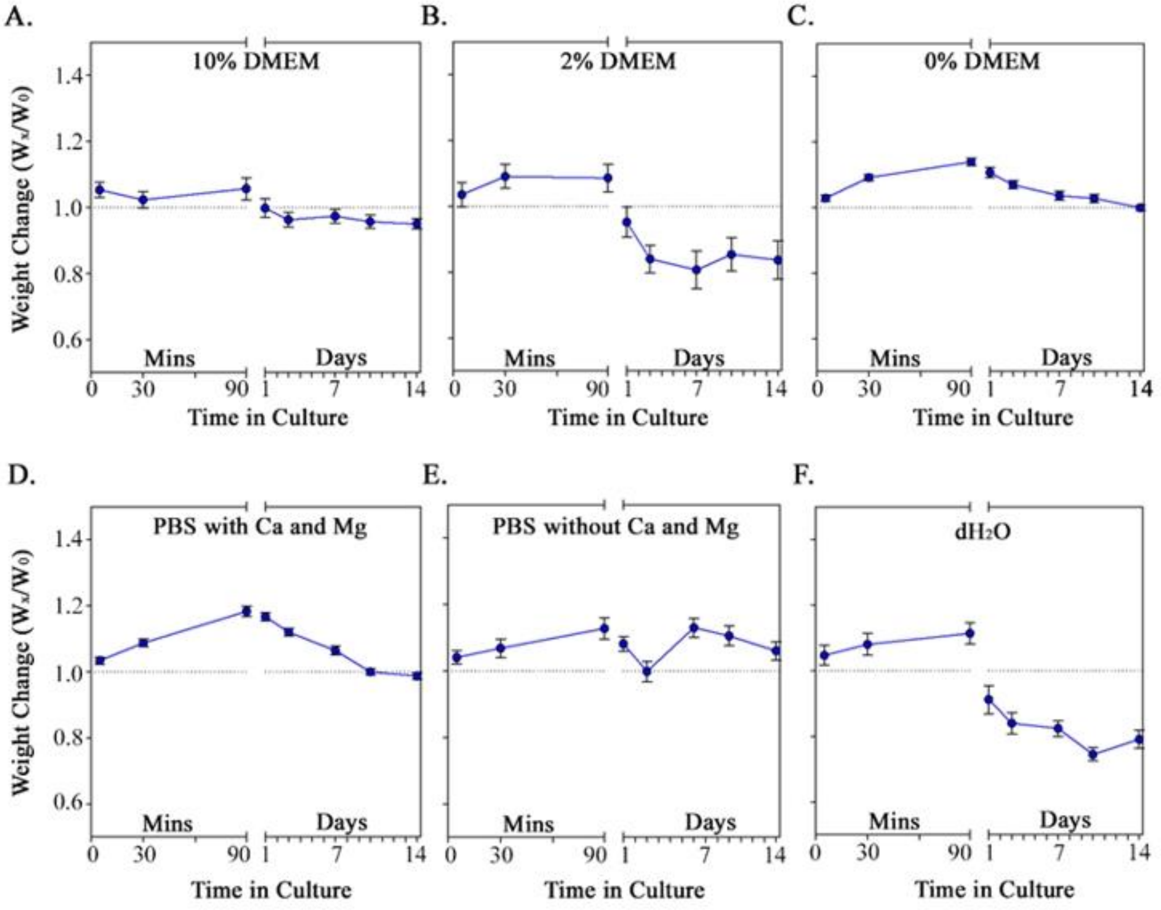

Figure S8. Weight fluctuation of 6:2 scaffolds during the first two weeks of culture. Weight of 4:2 scaffolds was measured at various times after printing (W_x) and normalized by initial weight (immediately after crosslinking, W_0) to yield a weight change ratio (W_x/W_0) for each scaffold. Scaffolds were cultured in Dulbecco's Modified Eagle Medium (DMEM) containing (**A**) 10%, (**B**) 2%, or (**C**) 0% fetal bovine serum (FBS), (**D**) PBS with Ca^{2+} and Mg^{2+}, (**E**) PBS without Ca^{2+} and Mg^{2+}, and (**F**) deionized water (dH_2O). Dotted line indicates initial weight (W_0/W_0). Data represents n = 4 with at least three technical replicates.
